# Bioinspired flow sensor enables underwater robots to estimate motion and detect flow structure

**DOI:** 10.1126/sciadv.aed2847

**Published:** 2026-06-03

**Authors:** Myungsun Park, Allyson E. Chen, Jacobo Cervera-Torralba, Michael T. Tolley, Geno Pawlak

**Affiliations:** ^1^ Department of Mechanical and Aerospace Engineering, University of California San Diego, La Jolla, CA, USA.; ^2^ Material Science and Engineering Program, University of California San Diego, La Jolla, CA, USA.; ^3^ Scripps Institution of Oceanography, University of California San Diego, La Jolla, CA, USA.

## Abstract

We address the challenge of sensing self-motion and environmental information by autonomous underwater vehicles. To overcome the limitations of conventional sensing methods in terms of size, cost, and environmental restrictions, previous work has investigated biomimetic flow sensors. Challenges in application of those biomimetic sensors include measuring complex locomotion of the vehicles and detecting external flow structure during the motion, all within a compact form suitable for a small vehicle. To address the challenges, we present a small and lightweight soft magnetic hair flow sensor that can measure the speed, direction, and oscillation of flow based on its mechanical deflection. We tested the ability of these sensors on underwater robots to estimate their forward, lateral (angular) speeds and orientations. While the robots were swimming, the sensors could also detect the wake behind an upstream object by identifying its characteristic frequency. The bioinspired hydrodynamic sensing, as demonstrated with the proposed sensors in this article, could allow adaptive and efficient underwater exploration.

## Teaser

Bioinspired flow sensors mounted on underwater robots enabled motion estimation and detection of an upstream wake.

## INTRODUCTION

Marine animals feel water flow using hydrodynamic sensors (*1*, *2*). A common biological sensing mechanism converts the deflection and vibration of soft protruding structures induced by the motion of the animals and by external flow structure, to electrical signals. For example, fish have an array of hair-like gelatinous structures for measuring speed and pressure gradients (*2*, *3*), and seals have long whiskers to detect and follow nearby animals (*4*, *5*, *6*). This sensing modality allows the animals to monitor their motion through water and also provides information about their environment that cannot be captured by vision, such as flow patterns and the presence of objects in low light or at distance (*7*). This ability enables many remarkable behaviors such as self-organized undulatory motions (*8*), alignment of the body along incoming vortices (*9*), predation by trail-tracking (*4*), and schooling (*10*), and it persists even in animals with poor vision or in dark or murky environments (*11*).

The state-of-the-art technologies for sensing flow are generally lacking similar perception in autonomous underwater vehicles (AUVs). The standard method for measuring underwater velocity is based on acoustic sensors such as the Doppler velocity log (DVL) or acoustic Doppler current profiler (ADCP). DVLs and ADCPs are generally too large and heavy [66-mm diameter and 170 g for the smallest device in the market; (*12*)] to be used for small-scale vehicles and are also expensive [>$10,000 (US) for the most basic options] (*13*–*15*). They also cannot measure close to the surface of a vehicle because they require a blanking distance for transmission and reception of the pulses [5 cm as the smallest option; (*12*)], which limits their ability to sense the flow-structure interaction for a vehicle. Particle image velocimetry has been used to measure flow close to the surface (*16*, *17*); however, it is not practical to use on-board or in real time. Vision-based, pressure-, and electromagnetic (*18*) sensing principles have also been used but they are sensitive to environmental conditions such as visibility, depth, and conductivity. A small, light, and inexpensive sensor technology that directly measures the flow at the surface of a vehicle would resolve those limitations and could complement or substitute existing technologies for some applications.

Researchers have been inspired by biological sensors to solve challenges in measuring flow for underwater autonomy. Two notable approaches to bioinspired flow sensing are distinguished by the types of sensors used. The first mimics the distribution of biological pressure sensors on the surface of marine animals, making use of an array of pressure sensors along the vehicles. This approach is based on the Bernoulli equation for unsteady flow, which describes the variation of hydrodynamic pressure next to a body given its motion relative to the fluid (i.e., linear and angular velocity) (*19*–*23*). Despite the simplicity of using off-the-shelf pressure sensors, there is a fundamental challenge: Because these sensors do not directly measure the desired quantity (flow velocity), the complex relationships between pressures and the velocities that depend on the geometry of the body must be resolved using a large amount of data collected by a number of sensors (*20*, *22*). According to the previous studies that have examined these relationships (*22*, *24*–*26*), the optimal number and placements of the sensors to estimate the velocities depend on the types of motions involved (e.g., rectilinear or turning) (*22*) and vorticity of the incoming flow (*26*), and thus preclude a simple implementation of the technology.

**Fig. 1.**
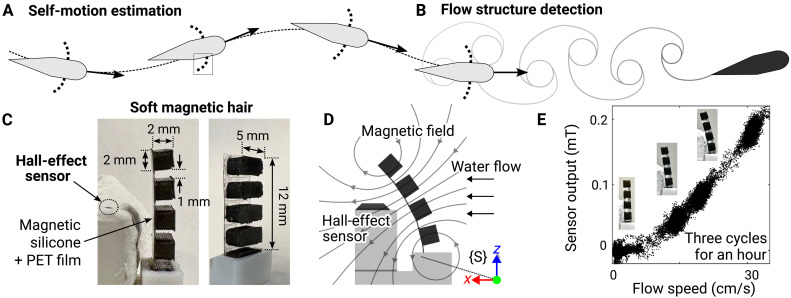
Biomimetic soft magnetic hair sensor for measuring flow. (**A**) Self-motion tracking of underwater vehicles and (**B**) detection of surrounding flow structure using soft magnetic hair flow sensors. (**C**) Image of the soft magnetic hair with dimension, and (**D**) illustration describing the magnetic sensing mechanism and sensor frame {*S*}. (**E**) Sensor output to unidirectional flow in *x* direction (three cycles). Inset images show the magnetic hair deflected at different velocities.

The second approach to bioinspired flow sensing mimics biological mechanoreceptors that directly measure the flow (Fig. 1) (*27*–*33*). Besides measuring the overall velocity that is related to self-motion (Fig. 1A), these biomimetic sensors also aim to detect fluctuating components in the flow that can provide additional information about the environment, such as fluctuations due to the presence of nearby objects (Fig. 1B) (*34*, *35*). Previous work has investigated various sensing principles (*36*–*43*) and optimized designs for desired mechanical properties (*43*–*47*) to enhance sensitivity, bandwidth, modularity, and noise reduction (*48*–*50*). Building on these studies, recent work has begun to use such biomimetic sensors for estimating the motion of underwater vehicles, including a fairing structure with a strain gauge to estimate forward velocity (*51*, *52*) and an elastomeric “tentacle” whose deformation was captured by a camera to estimate both speed and direction (*43*). These studies demonstrated the potential of underwater robots using biomimetic flow sensing to track their motion.

However, many challenges still remain to achieve a level of perception equivalent to biological systems. First, the sensors should have a simple design for practical, low-cost implementation (*53*). Second, they should distinguish the direction of motion, providing sufficient information in small numbers to track the complex underwater locomotion such as undulatory swimming of fish (*54*). Third, their mechanical properties should allow the detection of oscillations in external flow alongside the self-motion of the vehicle.

In this work, we address these challenges in self- and environmental sensing of AUVs by developing a soft magnetic hair flow sensor (Fig. 1C) that can directly measure flow in a compact design. For tracking the forward and transverse motion of underwater vehicles, we adopt an asymmetrical design for the sensor to distinguish the orientation of the flow. To achieve sufficient sensitivity and dynamic properties (hysteresis, etc.) for measuring both the overall velocity and oscillation of the surrounding flow, we integrate two distinct materials to serve separate roles as a signal generator and a mechanical support. To investigate all the functionalities of the sensors and their applicability to a real-world scenario, we implement them on a swimming robot and discuss the potential challenges for long-term missions in marine environments.

## RESULTS

### Biomimetic soft magnetic hair sensor for measuring flow

In this work, we developed a biomimetic mechanoreceptive sensor that responds to the flow adjacent to the surface of underwater vehicles to infer useful information for locomotion and navigation. This information includes measurement of the self-motion of the vehicles (Fig. 1A) and detection of external flow structure (Fig. 1B). A single unit of the proposed sensing system consisted of two components, a soft magnetic hair and a commercially available Hall-effect sensor (Fig. 1C). The soft magnetic hair was composed of an array of cuboids that were composites of magnetic particles (neodymium-iron-boron, NdFeB) and platinum-cured silicone (Dragon Skin 30, Smooth-On, Inc.) mounted on a polyethylene terephthalate (PET) film. The PET film served as a backing layer to support deformation against the drag induced by the flow. When water flow deflected and oscillated the soft magnetic hair, the Hall-effect sensor converted the change of magnetic field into an analog voltage signal (Fig. 1D). Details about the materials and the fabrication process are provided in Materials and Methods and fig. S1.

We designed the magnetic hair to have desirable mechanical properties to respond to both static and dynamic stimuli generated by fluid flow. An ideal mechanoreceptive flow sensor needs to have minimal plastic deformation, hysteresis, and time-dependent deformation (creep). In addition, it must be critically damped for spontaneous response and minimizing the effect of its intrinsic motion over the flow-induced displacements (*5*, *27*, *46*). To meet these criteria, we chose a backing PET film with Young’s modulus of 2 to 4 GPa and thickness of 100 μm, such that its properties dominated the mechanical response of the hair. More discussion on the material selection is presented in Discussion.

To characterize the static response of the sensor to unidirectional flow, we observed the output of the sensor in response to changes in the flow speed from 0 to 30 cm/s over three cycles for an hour. There was no drift in signal for the three cycles with negligible hysteresis (Fig. 1E). Time series data for the cyclic tests are presented in fig. S2. To qualitatively evaluate dynamic characteristics, we conducted a step relaxation test (displacing the end of the hair and releasing). The hair showed minimal creep, and its natural vibration was damped in water, even though it was not critically damped (movie S1). These mechanical properties of the hair allowed the accurate measurement of not only overall velocity but also dynamic oscillation of the flow, which we discuss in the “Detection of flow structure and estimation of motion in nonuniform flow” section, below.

### Orientation-dependent responses to directional flow

**Fig. 2.**
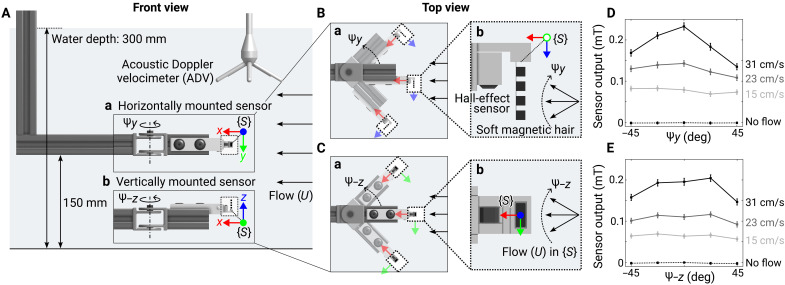
Orientation-dependent sensor response to directional flow. (**A**) Front view of experimental setup to characterize the sensor response to directional flow. The water flume generated a steady and uniform flow, whose speed was measured by an ADV as a reference. The rotating mount changed the orientation of the sensor to the flow. {*S*} indicates the sensor frame. (**B**) Top view of the horizontally mounted sensor to measure directional flow, defined by the angle $\psi_{y}$ in the *xz* plane of the sensor frame. Positive $\psi_{y}$ indicates counterclockwise rotation of the mount in the global top view (a) and clockwise rotation of the flow around positive *y* axis in the sensor frame (b). (**C**) Top view of vertically mounted sensor to measure directional flow, defined by the angle $\psi_{-z}$ in the *xy* plane of the sensor frame. Positive $\psi_{-z}$ indicates counterclockwise rotation of the mount in the global top view (a) and clockwise rotation of the flow around negative *z* axis in the sensor frame (b). (**D**) Sensor output to the flow with different speeds and directions ($\psi_{y}$). (**E**) Sensor output to the flow with different speeds and directions ($\psi_{-z}$).

Due to the asymmetric design of the soft magnetic hair, the speed and direction of the flow both influenced the response of the sensor. To describe the orientation of the sensor relative to flow, we defined the local frame of the sensor, {*S*}, centered at the base of the hair with the *x* axis directed toward the Hall-effect sensor and the *z* axis pointing along the hair (Fig. 1D). To characterize the effect of this relative orientation, we built an experimental setup in a water flume with a rotating mount for the sensor. By changing the angle of the mount in the unidirectional flow of the flume, we tested the sensor’s sensitivity to orientation. With the sensor placed on the side of the mount, the hair (the *z* axis of the sensor frame, {*S*}) was horizontal in the flume [Fig. 2A (a)]. Here, the flow varied relative to the sensor with angle $\psi_{y}$ around the positive *y* axis [Fig. 2B (b)]. With the sensor on the top of the mount, the hair was vertical [Fig. 2A (b)], and the flow varied with angle $\psi_{-z}$ around the negative *z* axis [Fig. 2C (b)] (see Materials and Methods and movie S2 for details).

These experiments showed that the sensor distinguishes flow direction in the *xz* plane (Fig. 2, B and D) more effectively than in the *xy* plane (Fig. 2, C and E). For flow direction varying in the *xz* plane, the sensor output was asymmetric to the flow direction (Fig. 2D). In this case, the output was larger when the magnetic hair (positive *z* axis of {*S*}) was aligned in the same direction with the flow ($\psi_{y}<0$) than in the opposite direction ($\psi_{y}>0$). On the other hand, for flow direction varying in the *xy* plane, the sensor output was primarily determined by the speed and was symmetric to the direction (i.e., the sensor did not distinguish whether the flow was angled to the right or to the left; Fig. 2E). The entire time series of the sensor outputs in Fig. 2 (D and E) are presented in fig. S3.

The sensors’ dependence on orientation provided guidance for selecting the number of sensors and their placements to enable the measurement of the motion of swimming robots. For example, we decided to place the sensors on the side of the robot to measure its angular velocity, assuming that the sensors would capture the yawing motion by making the magnetic hair deflect in the horizontal plane (*xz* plane). This reasoning allowed us to minimize the iterations for design and the number of sensors, providing two advantages of the proposed sensing system over other alternatives. We further compare the advantages and disadvantages of different sensing methods in Discussion and table S1.

### Estimation of swimming motion in uniform flow

**Fig. 3.**
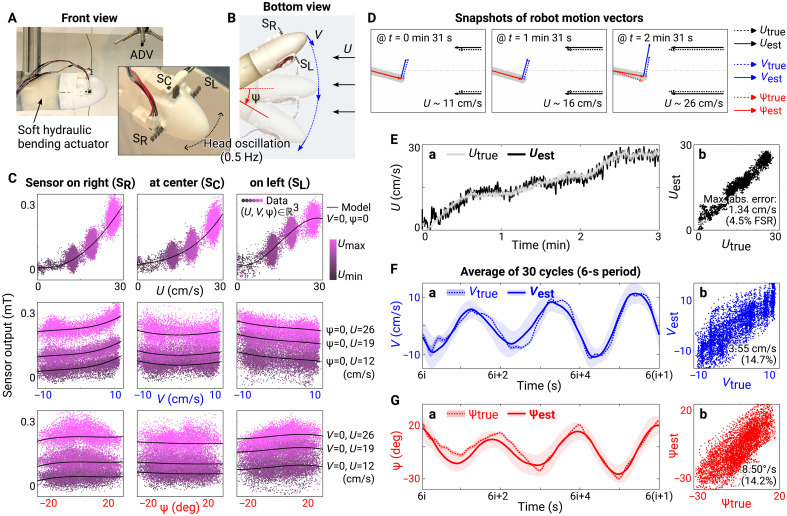
Estimation of swimming motion in uniform flow. (**A**) Experimental setup for simulating biological swimming motion using a soft underwater robot equipped with three magnetic hair flow sensors. (**B**) States (horizontal flow speed, *U*, lateral speed, *V*, and yaw angle, ψ, of the robot) defined to describe the dynamic system where the head of the robot was oscillating at 0.5 Hz in the uniform flow generated by the flume. (**C**) Sensor responses to the three states. Scattered data points are the measured outputs from the sensors with *U*, *V*, and ψ spanning 0 to 30 cm/s, −10 to 10 cm/s, and −20° to 20°, respectively. The increasing intensity of the colors indicates increasing speed of the flow (*U*) at the moment. The black curves depict the third-order polynomial model with two variables held constant, as indicated by the text on the right. (**D**) Visualization of true and estimated states of the oscillating soft underwater robot in uniform flows of different speeds. Dotted lines indicate the true states and solid lines indicate their estimations. (**E**) Time series (a) and comparison (b) of the true and estimated (est) *U* for 3 min. Text in each plot indicates the mean absolute estimation error and its percentage relative to the full-scale range (FSR) of the true values. (**F** and **G**) Time series (a) and comparison (b) of the true and estimated *V* and ψ for 3 min. The time series shows averages and standard deviations over 30 cycles.

Biological fish use their hair sensors to measure the speed and direction of flow next to their body while swimming (*2*, *3*). To demonstrate similar capabilities with our magnetic sensors, we attached them to the anterior part of a soft, underwater robot (*55*, *56*) fixed at a transverse plane but able to yaw its head (Fig. 3A). We then used the sensors to estimate the relative velocities and angles while the head of the robot rotated in flows of different speeds (13, 21, and 29 cm/s). The head of the robot oscillated with a varying amplitude of up to 55 mm at 0.5 Hz, driven by periodic bending of the body using a soft hydraulic actuator (see movie S3 and Materials and Methods for details on the experimental setup). We compare the motion of the robot to biological swimming in Discussion.

We determined the proper number and placement of the sensors to estimate the motion of the robot relative to the flow. We modeled the system to have three states: the speed of the horizontal flow (*U*), the lateral speed at the tip (*V*), and the yaw angle (ψ) (Fig. 3B), as the motion of the undulating body of a swimming fish can be decomposed into forward, lateral (sway), and angular (yaw) components (*57*). By conducting principal components analysis, we confirmed that the states were three-dimensional (3D) and each of the principal components varied in similar proportion (detailed results of the principal components analysis are presented in fig. S4). This system differed from the steady cases in the previous section, where we only considered the effect of horizontal flow speed (*U*) and the static angle (ψ), in that the relative velocity of the flow near the sensors now varied with the lateral speed (*V*) and the rotation of the body.

To resolve this coupled effect of the three variables, lateral speed (i.e., *V*), orientation (i.e., ψ), and the (effective) forward speed (i.e., *U*), we placed one sensor vertically at the center (S_C_ in Fig. 3A) and the other two horizontally on the sides of the head [sensor on the right (S_R_) and sensor on the left (S_L_)]. This placement was grounded on the observation from the previous section that the orientation influenced the signal only when the sensor was mounted horizontally (Fig. 2). Our hypothesis was that the sensor at the center would isolate the forward velocity (*U*) and the other two would disambiguate the magnitude (*V*) and direction (ψ) of the lateral velocity.

The sensor measurements supported the hypothesized observability of the states *U*, *V*, and ψ (Fig. 3C). Outputs from the three sensors increased with increasing horizontal flow speed (*U*), as expected from the previous test (Fig. 1E). The responses of the sensors to the lateral speed (*V*) and orientation (ψ) of the body, however, were distinct between the sensors. As the head was moving left (positive *V* in Fig. 3, B and C), the output of the sensor on the right (S_R_) increased due to the relative flow pushing the magnetic hair toward the Hall-effect sensor. At the same time, the sensor on the left (S_L_) was exposed to flow that pushed the hair away from the Hall-effect sensor, resulting in decreased output. When the head moved to the right (negative *V*), the outputs of the two sensors changed inversely. The shape of the sensor output curves with varying orientation (ψ) was similar to that observed in the steady experiment (Fig. 2, D and E). For the sensors on the sides, the outputs were asymmetric with respect to the flow direction. The outputs were larger when the magnetic hairs were aligned with the flow (negative $\psi_{y}$ in Fig. 2, B and D) compared to when they were opposite to it (positive $\psi_{y}$ in Fig. 2, B and D). The output of the sensor at the center also agreed with the result from the previous experiment, being less sensitive to the lateral motion than the sensors on the sides and symmetric to the direction.

We then estimated the states of the system using the sensor signals for 3 min, while the robot repeatedly oscillated its head with three different amplitudes and the horizontal flow speed increased from 0 to ∼30 cm/s (see fig. S4B). We used an extended Kalman filter with a third-order polynomial sensor model fitted with 12-min training data, including head rotation amplitudes that varied linearly and randomly, and horizontal flow speed increasing stepwise (see fig. S4A and Materials and Methods for details). We compared the true and estimated states at every time step in Fig. 3 (D to G) and movie S4. The mean absolute errors of the estimation for *U*, *V*, and ψ were 1.34 cm/s (4.5% of the full-scale range of the true values), 3.55 cm/s (14.7%), and 8.50°/s (14.2%), respectively. The errors were higher for the lateral speed (*V*) and yaw angle (ψ), compared to the horizontal speed (*U*). This can be explained by two aspects. First, the sensor at the center was not sensitive enough to contribute to estimating yawing and lateral motion (Fig. 3C). Second, the other sensors on the sides were also less sensitive to the relevant directional flow compared to the horizontal flow, as shown by the smaller variation in the sensor signals in response to individual state changes (black curves in Fig. 3C).

By comparing this estimation performance to cases with one or two of the three sensors omitted using the same extended Kalman filter, we found that the performance degraded when fewer sensors were used (fig. S5). The accuracy of the forward speed estimation decreased as the number of sensors decreased. For estimating the lateral velocity (*V*) and the yaw angle (ψ), the effect was especially substantial when only the center sensor (S_C_) was used because it could not distinguish the direction of lateral motion.

To understand the variability of the estimation performance with different numbers and placement of the sensors, we conducted observability analysis (*58*) for each combination of the sensors (fig. S6). The observability matrix ($O=\left[H_{k};H_{k}F;H_{k}F^{2};\ldots \right]$) were always full rank during the experiment. The condition numbers of the observability matrices with only one sensor were larger than with more sensors by three orders of magnitude. When either of the two sensors on the side (S_R_ or S_L_) was not used, fluctuation of the condition number for the oscillation of head became larger than when all of them were used due to increased ambiguity for the lateral motion. Further discussion on the relationship between estimation performance and sensor placement is in Discussion.

### Detection of flow structure and estimation of motion in nonuniform flow

**Fig. 4.**
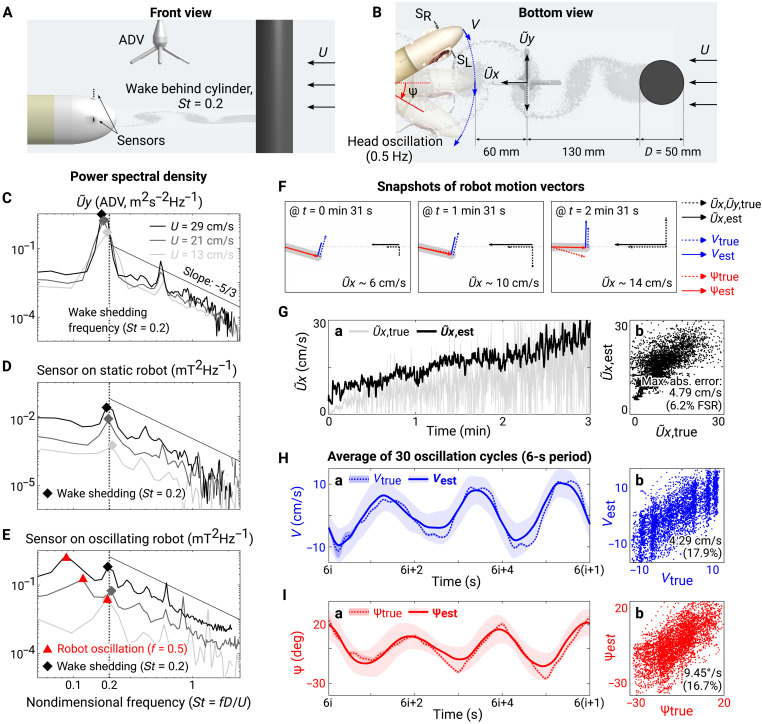
Detection of flow structure and estimation of motion in nonuniform flow. (**A**) Experimental setup for detecting a wake behind a cylinder using hair flow sensors. (**B**) States defined to describe the dynamic system while the head of the robot was oscillating at 0.5 Hz in the wake shedding behind the cylinder: speed of the upstream horizontal flow (*U*), streamwise component in the wake (${\tilde{U}}_{x}$,), cross-stream component in the wake (${\tilde{U}}_{y}$), lateral speed (*V*), and yaw angle (ψ) of the robot. (**C**) Reference energy spectra of the cross-stream component (${\tilde{U}}_{y}$) in the wake measured by ADV in nondimensional frequency (*St*). The vertical dashed line indicates the frequency of the wake (*St* = 0.2), and the solid line indicates the energy cascade in turbulent flow, with slope of −5/3. (**D**) Energy spectra measured by the sensor on the static head. (**E**) Energy spectra measured by the sensor mounted on the oscillating head. The red markers indicate the frequency of the head oscillation (*f* = 0.5 Hz). (**F**) Visualization of true and estimated states of the oscillating soft underwater robot in nonuniform flows of different speeds. Dotted lines indicate the true states, and solid lines indicate their estimations. (**G**) Time series (a) and comparison (b) of the true and estimated ${\tilde{U}}_{x}$ for 3 min. Large variance in the true value reflects the high turbulence in the wake. Text in each plot indicates the mean absolute estimation error and its percentage relative to the FSR of the true values. (**H** and **I**) Time series (a) and comparison (b) of the true and estimated *V* and ψ for 3 min. The time series shows averages and standard deviations over 30 cycles.

Animals use their sensors not only to measure self-motion but also to assess their environment by detecting the frequency characteristics of flow structure in the surrounding fluid (*1*, *4*–*6*). To test the performance of the magnetic hair flow sensor in resolving external flow oscillations in addition to estimating the robot’s self-motion, we placed a circular cylinder (diameter: 50 mm) upstream of the sensors in a flume, which generated a repeating pattern of alternating signed vortices [Karman vortex street; (*59*)] (Fig. 4, A and B). The frequency of the shedding was determined by the upstream velocity (*U*) and diameter (*D*) for a cylindrical object, resulting in a Strouhal number (*St* = *fD*/*U*) of around 0.2 (*60*). We tested with three upstream velocities (13, 21, and 29 cm/s) and two mounting conditions for the sensors: First, the head was static so that the sensors only experienced the steady incoming flow, and second, the head oscillated at 0.5 Hz so that the sensor outputs were affected by the head motion, a more realistic condition. We used the acoustic Doppler velocimeter (ADV) behind the cylinder to measure the reference signals (movie S5).

The energy spectra of the magnetic hair flow sensor output showed that the sensors could detect the characteristic frequencies of the flow and could also distinguish the overall level of the flow kinetic energy. The vortex shedding frequency for the wake (*St* = 0.2; Fig. 4C) was identified by the hair flow sensors for both the static (Fig. 4D) and the oscillating (Fig. 4E) cases, as indicated by grayscale diamond markers. When the shedding frequency was close (about 0.55 Hz at *U* = 13 cm/s) to the oscillation frequency of the head (*f* = 0.5 Hz, indicated by red triangle markers in Fig. 4E), the two peaks merged. However, the shedding frequency could still be identified after removing the head oscillation component from the signal using a band-stop filter with a stopband of 0.49 to 0.51 Hz (fig. S7). Further discussion on the decoupling of the signals is provided in Discussion.

For all the measurements, the overall level of the energy spectra was higher for larger upstream velocities, demonstrating that the devices were able to measure magnitudes of oscillations in large bandwidth. This suggests the potential use of the sensor for measuring turbulent kinetic energy through characterizing the transfer function that relates the sensor’s spectral response to flow velocity. In the high-frequency region, the energy densities decreased linearly with frequency following a slope of −5/3, consistent with the canonical energy cascade for turbulent flow (*61*). Details on the methods for computing the energy spectra and processing the data are described in Materials and Methods.

Furthermore, we examined whether the sensors could estimate the motion of the robot under this nonuniform flow condition (Fig. 4, F to I, and movie S6). We used the same extended Kalman filter and the sensor model fitted with the data collected in uniform flow (Fig. 3C and fig. S4A) and tested the performance for the same motion pattern of the head in the nonuniform wake. In this way, the cross-stream component in the wake (${\tilde{U}}_{y}$) can be regarded as an unknown perturbation in addition to the streamwise flow speed (${\tilde{U}}_{x}$), lateral speed (*V*), and yaw angle (ψ) of the robot as states to estimate.

The maximum absolute errors in all three states increased compared to the uniform-flow case. First, the filter overestimated the streamwise flow speed (${\tilde{U}}_{x}$), which may partly reflect actual difference in velocity: Because the sensors were located downstream of the reference device (ADV) and laterally offset from the cylinder during oscillation, the local flow speed at the sensors could have been higher than that measured by the ADV. The error may also include effects of cross-stream flow, which could have induced additional deformation of the sensors. Second, the standard deviation of the estimated lateral speed (*V*) and yaw angle (ψ) increased, likely due to the oscillating flow in the perturbation.

### Application on free-swimming robotic fish

**Fig. 5.**
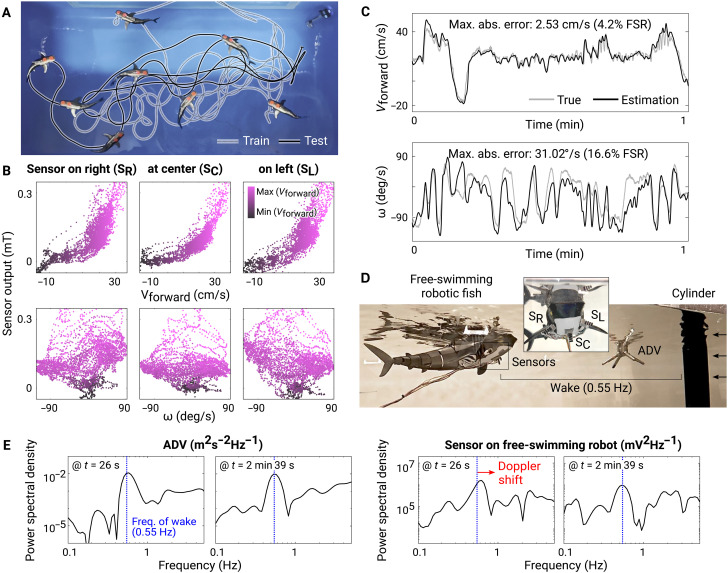
Application on free-swimming robotic fish. (**A**) Free-swimming robotic fish in the pool. (**B**) Sensor responses to forward and angular velocities. The intensity of the colors represents the magnitude of the forward velocity at the moment. The forward velocity was calculated as the magnitude of the velocity vector projected in the forward direction heading from the rear marker to the front marker. (**C**) Time series of the true and estimated states for 1 min of the test. Text in each plot indicates the mean absolute estimation error and its percentage relative to the FSR of the true values. (**D**) Robotic fish swimming against the wake behind a cylinder in water flume. (**E**) Instantaneous energy spectra of the measurements by ADV and hair flow sensors at two moments (units: hertz). The vertical dashed line (blue) indicates the frequency of the wake behind the cylinder (0.55 Hz).

In the previous sections, we investigated the two functionalities of the proposed sensor for underwater robots: estimation of self-motion and detection of surrounding flow structure. Building on these results, in this section, we discuss a realistic implementation of three sensors installed on a free-swimming robotic fish (2.4G Remote Control Shark Toy, Coodoo), with one at the center and two on the sides. First, to test the ability to estimate self-motion, we operated the robotic fish to swim a random trajectory in the pool (Fig. 5A). Meanwhile, we recorded its forward ($V_{\text{forward}}$) and angular velocity (ω) calculated from the positions of the two markers along with the sensor measurements over 170 s. Details on the operation of the robot, tracking markers, postprocessing of the data, and comparison between training and test datasets are provided in Materials and Methods and fig. S8.

The responses of the sensors to forward and angular velocities (Fig. 5B) were similar to their responses to the speed of the incoming horizontal flow (*U*) and lateral velocity of the head (*V*), respectively, in the previous experiment (Fig. 3C)—outputs from all the sensors increased as the forward velocity increased, and the two sensors on the sides (S_R_ and S_L_) responded inversely to the direction of the angular velocity, while the other at the center (S_C_) showed symmetric response to the direction.

As in the previous experiment, we estimated the states of the motion ($V_{\text{forward}}$, ω) by using an extended Kalman filter with a third-order polynomial sensor model. We used the first 110 s of the data as the training set to fit the model and then tested the estimation performance with the remaining 60 s of data (fig. S8). The mean absolute errors of the estimation for the forward and angular velocities were 2.53 cm/s (4.2% of the full-scale range of the true values) and 31.02°/s (16.6%), respectively. The swimming motion of the robot during the test is presented with the true and estimated states along with the measurement by the sensors at each moment in movie S7.

We then operated the robotic fish in the flume to swim against the flow behind the cylinder to test the ability to detect the structure of surrounding flow (Fig. 5D). The upstream velocity of the flow in the flume was constant at about 13 cm/s throughout the test, which resulted in the wake with a frequency of 0.55 Hz behind the cylinder. We tested various swimming patterns with the robotic fish, including those with the following: minimal and exaggerated lateral movements, a frequency matching or higher than that of the wake, and motion toward upstream and drifting downstream (movie S8). For this experiment, we computed the energy spectra of the sensor and the ADV measurements at every time step using a wavelet transformation (Fig. 5E) (*62*, *63*). Details on the methods for computing the energy spectra in real time are explained in Materials and Methods.

The frequency of the wake was intermittently captured in the energy spectra of the sensor measurements depending on how the robotic fish was moving (fig. S9 and movie S8). When the robot was moving with minimal lateral displacement or oscillating at a much higher frequency than the wake, a peak near the wake frequency (0.55 Hz) in the spectrum was better distinguished than for other motions. When the robotic fish had a relative motion toward or away from the cylinder (source of wake), the peak in the sensor signals was shifted from 0.55 Hz to larger or smaller value, respectively (Doppler shift) (Fig. 5E).

### Biofouling effect on sensor response

**Fig. 6.**
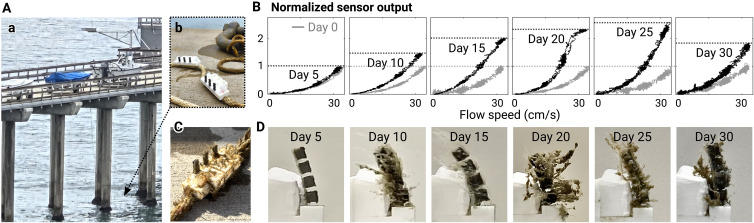
Biofouling effect on sensor response. (**A**) Deployment at Scripps pier (a). Six magnetic hair samples were tied to a rope with a weight to submerge in the water (b). (**B**) Output of the sensors to unidirectional flow, before (gray curves) and after (black curves) days of deployment. The output from each sample was normalized by the maximum output at about 30 cm/s before the deployment. The sensor became more sensitive with fouling, likely due to the added drag of attached biological structures. (**C**) Three of the samples that had been submerged for 15 days. (**D**) Images of biofouled magnetic hairs submerged in ocean water for up to 30 days.

To investigate the possibility of using the proposed sensors in the ocean over a prolonged period, we tested the effect of biofouling on the sensor measurements. We fabricated six samples of the magnetic hair and measured their responses to a cycle of unidirectional flow ranging from 0 to about 30 cm/s. We then submerged the samples in ocean water off of Scripps Pier, La Jolla, CA, for up to 30 days (Fig. 6A). We retrieved one sample every 5 days and measured the response again in the same setup (Fig. 6B).

Biofouling was observed on all of the samples (Fig. 6C). All the fouled samples remained magnetic and produced increasing signal output as speed of flow increased without hysteresis, as before the deployment. However, the sensitivity of the sensors was altered as a result of fouling—sensors generally produced larger signals than before, as the increased contact area against the flow caused greater drag (Fig. 6, B and D). The change of the signal was broadly proportional to the extent of fouling, where the most fouled samples (after 20 or 25 days) resulted in the largest deviation from the original curve. The fouling effect, however, did not increase indefinitely over time. For example, the sample submerged for 30 days showed less fouling than the ones after 20 or 25 days and accordingly resulted in less deviation from the original output curve.

We also confirmed that salinity did not affect the sensors by comparing the measurements while being towed at controlled speeds (10, 20, and 30 cm/s) in fresh and saline (3.5%) water (fig. S10). Based on these results, we will discuss strategies to address fouling effects associated with prolonged deployment in the ocean in Discussion.

## DISCUSSION

### Advantages over existing technologies and current limitations

The sensing principle of the proposed sensor, using mechanical deformation induced by drag, has advantages over other sensing principles that have previously been used for similar applications. Compared to acoustic sensors, which are the state-of-the-art technology for measuring velocities underwater, the most important advantage for the proposed sensor is its reduced size. The smallest DVL in the market has a diameter of 66 mm, height of 25 mm, and requires at least 50 mm of clearance between the device and the sampling volume (*12*). The proposed sensor has a width of 5 mm and a height of 12 mm, and can measure the flow exactly at its position, which can provide direct information for understanding the interaction between flow and the structure where it is mounted. Cameras, pressure sensors, and electromagnetic sensors, which have advantages in size over acoustic methods, are often restricted by environmental conditions, such as visibility, depth, and conductivity, none of which affect the proposed sensor.

The proposed magnetic hair flow sensor also addressed many of the practical challenges in the existing methods for measuring flow near underwater vehicles or for estimating their motion. A sufficient number of pressure sensors can theoretically represent the motion of a moving object in water and has been shown to have good performance in previous work (*15*, *20*–*26*). However, pressure-based methods rely on complex models to relate velocities to pressures measured at many locations. The proposed sensor, which directly measures velocities, uses a relatively simple model and requires only three (or fewer) sensors. Conventional strain gauges required additional measures to suppress noise from turbulence (*51*, *52*). This was not a problem for the flow sensor featured here due to mechanical properties of the soft materials. These mechanical properties, including elasticity, minimal hysteresis, and creep, also enabled the sensing modality to detect the frequency of external flow, in addition to measuring the mean velocity of an underwater vehicle.

The current system also has some limitations. The deformation-based sensing principle cannot avoid effects of physical disturbances such as unexpected contact, and is also susceptible to fouling, as we observed. As drag is proportional to the square of velocity, the sensitivity of the sensor is worse at smaller velocities. This nonlinearity may limit the accuracy of the system; however, there is still room for improvement by optimizing the geometry of the hair (relating velocity to displacement) and placement of the Hall-effect sensor (converting displacement to voltage). This discussion is summarized in table S1 in the Supplementary Materials.

### Material selection and potential design improvement for soft magnetic hair

To fabricate the magnetic silicone for the soft magnetic hair, we mixed NdFeB particles and Dragon Skin 30 at a 10:1 volume ratio (Fig. 1C and fig. S1). We chose the mixing ratio to produce sufficient remanence to be measured by the Hall-effect sensor, based on previous work reporting the effect of these parameters on remanence (*64*). With less NdFeB, the magnetic hair still functioned but resulted in less sensitivity and lower resolution. When we used more, it was difficult to pour the mixture into the rectangular slots of the mold due to an increase in viscosity.

Future work could improve the current design for increased sensitivity and scalability. As its width (5 mm in current design) does not affect the sensitivity in theory, we could reduce the size of the hair to place the sensors more densely. The effects of changing the height require further investigation, as shorter hair will result in less sensitivity; however, it may include more information about interaction between the surface of robots and fluidic environment.

### Comparison to biological swimming motions

We envision the proposed sensors being applied to a wide range of underwater robotic systems including, but not limited to, ones that mimic biological locomotion to propel. Here, we are especially focused on the swimming motion of fish, involving continuous or intermittent yawing in addition to forward and lateral displacements. We described this motion and the associated fluid-structure interaction at the vicinity of the sensors in terms of two velocities (*U* and *V*) and an angle (ψ), and simulated the motion using the two experimental setups.

In the first setup with a hydraulic soft bending actuator, all three motion variables were controllable so that we could characterize the response of the sensors to them in both the time and frequency domains (Figs. 3 and 4). We covered a wide range of motions up to 30 cm/s of forward speed, 10 cm/s of lateral speed, and 30° of yawing angle. This resulted in an *St* between 0.17 and 0.33 (peak-to-peak amplitude of ∼100 mm at frequency of 0.5 Hz), which falls within the range observed for efficient swimming of fishes (0.2 to 0.4).

In the second experimental setup, we used a free-swimming robot to consider unsteady and irregular swimming motion, covering a variety of motions including starting, stopping, moving backward, and wide and tight turning (Fig. 5 and movies S7 and S8). Here, we confirmed that the functionalities of the sensors shown in the previous experiments, measuring velocities and detecting flow structure, were still valid under more realistic conditions. A previously unidentified functionality revealed in this free-swimming setup was the real-time detection of flow structure (Fig. 5, D and E; fig. S9; and movie S8). Compared to the results from the controlled setup (Fig. 4), the free-swimming experiments suggested a much more practical application of the sensing modality to any underwater robotic systems regardless of the complexity of their self-motion.

### Effect of sensor placement on accuracy

We compared the estimation performances of the motion of the robot in a uniform flow for different numbers and locations of sensors. We found that sensors placed on the sides were crucial not only for accuracy (fig. S5) but also for robustness in estimation as quantified by the condition number of the observability matrix (fig. S6). These results help explain the distribution of flow sensors observed in nature. For example, fish have a lateral line, which is an array of sensors extending from the head along the side of their body, while seals have whiskers distributed around their muzzle and along the sides of the head.

### Decoupling self-motion from external flow

The results on flow detection using the sensors (Figs. 4, D and E, and 5E) raise questions about strategies for the operation of the vehicle and the processing of sensor signals to decouple the self-motion from external flow information. In this work, we proposed two approaches to address these issues. First, the external flow structure could be reliably detected by operating the vehicle at a frequency much higher or lower than that of the flow (movie S8). Second, even when the robot oscillated at a frequency similar to that of the flow, the peak associated with the flow oscillation could be identified by filtering out the robot’s operation frequency (fig. S7).

Future work building on these observations could enhance the practical applicability of the proposed sensor for various underwater systems. First, as we can characterize the sensor signals associated with the robot’s motion, we can apply more sophisticated filtering to better isolate the signal of interest than removing a single frequency band. Second, insights into optimal robot operation may be drawn from nature by examining how animals hover near objects they want to probe and how they integrate inputs from multiple sensing modalities. This approach may motivate the use of external sensors, such as an inertial measurement unit or egocentric cameras, to measure motion in a global frame and complement the proposed flow sensor, which measures relative motion between the robot and its environment. This integration would likely enable accurate self-motion estimation and flow structure detection in complex underwater environments, including directional currents, waves, or local eddies, as well as in the presence of other animals or obstacles.

### Addressing fouling

We observed that the proposed sensor was able to operate with continuously changing sensitivity for at least a month in ocean water (Fig. 6 and fig. S10). As the change in sensitivity did not solely depend on the time since the deployment, we need some strategies for calibration of the system for prolonged missions. A simple solution could be to carry out a periodic self-calibration. For example, we could compare the integral of the velocity estimation by the sensors to a known reference trajectory during predefined operation of the vehicle. This approach, however, is not applicable if the task does not allow such predefined operations or, more importantly, when there is additional drag induced on the vehicle by external current, etc. In this case, we could use independent position measurements as references, such as cameras, pressure sensors, inertial measurement units, or a combination of these. We could also estimate the extent of fouling and the resulting mechanical properties, such as mass, stiffness, or damping, which influence the measured sensor signal, through spectral analysis—for example, by monitoring the natural frequency of the soft magnetic hair. Future work could also study how the fouling evolves over time by testing more samples for a longer time than those presented here. The model, however, may include a large uncertainty due to various factors such as sea state, water properties, and ecological conditions.

## MATERIALS AND METHODS

### Fabrication process of soft magnetic hair

We used a 3D-printed mold to cast the mixture of NdFeB particles (>99.9%, 400 mesh, Nanochemazone) and uncured silicone (Dragon Skin 30, Smooth-On, Inc.) to be magnetic composites attached to the backing PET film. We first printed the mold with five rectangular through-slots (Bambu Lab A1, Bambu Lab) and covered the bottom of the mold with a PET film coated with heat-activated glue and silicone adhesive (Sil-Poxy, Smooth-On, Inc.) (fig. S1A). We poured the mixture of NdFeB particles and silicone (volume ratio of 10:1) in the slots and covered the top of the mold with another PET film. At this point, the NdFeB particles were oriented in arbitrary directions and did not generate a net magnetic field (fig. S1B). We then placed the mold between two neodymium magnets (N52, diameter: 1 inch, thickness: 0.5 inch, McMaster-Carr) using 3D-printed holders and waited for 24 hours (fig. S1C). After 24 hours, the mixture was cured and the NdFeB particles inside were aligned, forming solid magnetic domains attached to the PET film on the bottom. We separated them from the mold (fig. S1D), trimmed the edges of the film, and assembled them with a 3D-printed mount to finish the fabrication of the soft magnetic hair (fig. S1E).

### Experimental setup in water flume

We tested the proposed sensor in a water flume to generate uniform horizontal flow with controlled speed (*U* in Fig. 2A). The main channel of the flume had a square cross-section with a width of 600 mm and was filled with water to a depth of 300 mm. We placed the sensor at mid-depth in the channel to make sure that the sensor measured uniform flows without wall effects. The mount for the sensor had a rotating joint at the end so that we could change the orientation of the sensor to the flow.

We selected three target velocities (13, 21, and 29 cm/s) for the experiments. To verify the flow velocity, we used an ADV (Vectrino, Nortek). We placed the ADV probe 100 mm away in horizontal and vertical directions from the sensor so that the ADV sampling volume was close to the sensor but not affected by associated flow disturbances.

### Experimental details for estimation of oscillating motion of underwater robot

To examine whether the proposed sensors could estimate dynamic states in biological swimming that involved forward and lateral (yawing) motions, we used the anterior part of a modular soft underwater robot fixed at a transverse plane to mimic the undulating motion of fishes (*55*, *56*). The anterior part consisted of a soft hydraulic bending actuator and a 3D-printed head. The actuator was oscillating relative to the fixed plane at a constant frequency of 0.5 Hz throughout the experiment, with varying amplitude controlled by a custom hydraulic control system (*65*). To measure the ground truth of the lateral speed of the head (*V*) and the yaw angle (ψ) of the robot, we attached two markers on the bottom of its head and recorded the video from the bottom to track the positions of the markers. The oscillating motion of the robot is presented in movie S3. We collected 12-min sets of data for identification and 3-min sets for testing of the system (fig. S4). ADV and sensor measurements were collected at 25 Hz.

### System identification for implementation of extended Kalman filter

We used an extended Kalman filter (*66*) to estimate the states (speed of the horizontal flow, *U*, lateral speed of head, *V*, and yaw angle, ψ) with consideration of the continuous propagation of the states and nonlinear response of the sensor signals (z) to the states (Figs. 1E; 2, D and E; and 3C). We modeled the propagation of the states as a second-order discrete-time dynamics from time step k to k+1 in a linear form

X(k+1)=FX(k) (1)

by defining constant state transition matrix *F* and internal state

X(k)=[U(k−1);V(k−1);ψ(k−1);U(k);V(k);ψ(k)] (2)

We modeled each of the measurements by the three sensors as a scalar-valued third-order polynomial in the state variables *U*, *V*, and ψ without coupling terms

$$\begin{array}{c} z_{i}=h_{i}\left(U,V,\psi \right)=a_{0}+a_{i1}U+a_{i2}U^{2}\\ +a_{i3}U^{3}+a_{i4}V+\ldots +a_{i9}\psi^{3}\left(i=1,2,3\right) \end{array}$$ (3)

to describe the nonlinear behavior of the sensors (Fig. 3C) while remaining differentiable for use in the Kalman filter. The models were approximated in linear form at each time step *k* as

$$z\left(k\right)=h\left[U\left(k\right),V\left(k\right),\psi \left(k\right)\right]\approx H\left(k\right)\left[U\left(k\right);V\left(k\right);\psi \left(k\right)\right]$$ (4)

by defining measurement matrix H(k) as a Jacobian of the nonlinear function

$$H\left(k\right)=\partial h/\partial x{|}_{x=x\left(k\right)}$$ (5)

where x=[U;V;ψ].

To identify the state transition matrix *F* and the measurement matrices H(k), we used 12-min training data (fig. S4A) collected before the actual test that covered the same ranges of the states.

### Computation of energy spectra for detection of flow structure

To investigate the frequency responses of the ADV and the proposed magnetic hair flow sensors to surrounding flow over a period of time (Fig. 4, C to E), we calculated the Fourier spectra for 4-min segments of data measured by the devices for three upstream velocities (13, 21, and 29 cm/s). Spectra were obtained using the pwelch function in MATLAB (R2025a, MathWorks), which implements Welch’s method for reducing noise by dividing the data into multiple windowed segments and averaging. We used 20-s-long windows, overlapped by 50% with a Hamming window used to reduce the spectral leakage. We also removed the noise floor for the signals assuming white noise. The noise level for each device (ADV and proposed sensors) was identified as the median of the power spectral densities in large frequencies (*St* > 3.5) with the lowest upstream velocity (13 cm/s). To resolve the merging peaks for robot oscillation and vortex shedding, we used a band-stop filter with a stopband of 0.49 to 0.51 Hz.

To test the ability of the proposed magnetic hair flow sensors to detect flow structure of the surrounding flow in real time (Fig. 5E and fig. S9), we conducted a wavelet transform. We used the cwt function in MATLAB with the Morlet wavelet for frequencies between 0.1 and 5 Hz.

### Experimental details for free-swimming robotic fish in the pool

To validate the performance of the proposed sensor in estimating the motion of a free-swimming robot, we operated the robotic fish to follow a random trajectory in the pool, including starting, stopping, straight swimming, wide and tight turning over 170 s (Fig. 5A). Time series and histograms of the training (the first 110 s) and testing (remaining 60 s) data are provided in the Supplementary Materials (fig. S8).

We placed two markers (diameter: 25.4 mm) on the robot to track its trajectory and calculate forward and angular velocities of the robot. We recorded video from above during the experiment and tracked the positions of the markers ($p_{1}$, $p_{2}$) using an open-source computer vision library (OpenCV). Using the heading direction (u) and the center point ($p_{c}$) of the robot

$$u=p_{2}-p_{1}$$ (6)

$$p_{c}=\frac{1}{2}\left(p_{1}+p_{2}\right)$$ (7)

the forward ($V_{\text{forward}}$) and the angular (ω) velocities were derived as

$$V_{\text{forward}}=\frac{dp_{c}}{\mathrm{dt}}\cdot \frac{u}{|u|}≅\frac{{\mathrm{\Delta p}}_{c}}{\Delta t}\cdot \frac{u}{|u|}$$ (8)

$$\omega =\frac{d\psi }{\mathrm{dt}}≅\frac{\Delta \psi }{\Delta t}=\frac{\text{atan}\frac{u\left(k+1\right)\times u\left(k\right)}{u\left(k+1\right)\cdot u\left(k\right)}}{\Delta t}$$ (9)

where ψ and Δt represent the heading angle and time interval between k- and (k + 1)-th data point.
