# Supplementary material for: Bioinspired flow sensor enables underwater robots to estimate motion and detect flow structure
Source: Sci Adv. 2026 Jun 3;12(23):eaed2847. doi: 10.1126/sciadv.aed2847 (PMC13232555; doi:10.1126/sciadv.aed2847)
Supplement: Supplementary file 1 — Figs. S1 to S10 Table S1 Legends for movies S1 to S8 [file sciadv.aed2847_sm.pdf]

Science Advances
AAAS

Supplementary Materials for

Bioinspired flow sensor enables underwater robots to estimate motion and detect flow structure

Myungsun Park *et al.*

Corresponding author: Myungsun Park, mypark@ucsd.edu

Sci. Adv. **12**, eaed2847 (2026)
DOI: 10.1126/sciadv.aed2847

The PDF file includes:

Figs. S1 to S10
Table S1
Legends for movies S1 to S8

Other Supplementary Material for this manuscript includes the following:

Movies S1 to S8

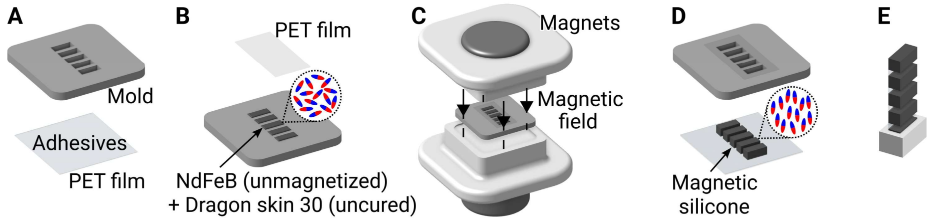

Fig. S1. Fabrication process of soft magnetic hair.

(**A**) 3D-printed mold covered by a Polyethylene terephthalate (PET) film on the bottom coated with adhesives. (**B**) Mixture of neodymium-iron-boron (NdFeB) particles and uncured silicone (Dragon Skin 30, *Smooth-On, Inc.*) poured into the mold and covered by PET film on the top. (**C**) Mold placed between two neodymium magnets by 3D-printed holders. (**D**) Solid magnetic domains formed after the mixture was magnetized and cured after 24 hours, and separated from the mold. (**E**) Soft magnetic hair assembled with a 3D-printed mount after edges of the PET film were trimmed.

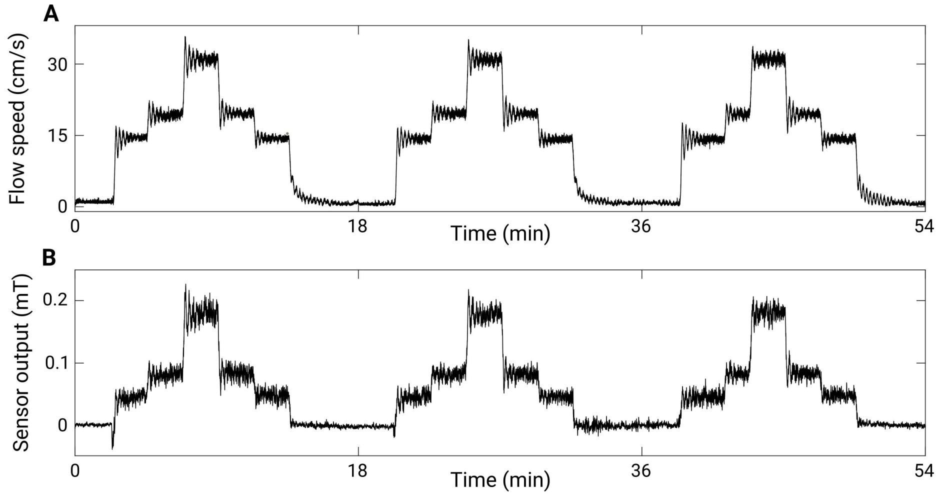

Fig. S2. Time series of cyclic unidirectional flow speed and corresponding sensor output. (**A**) Time series of unidirectional flow velocity measured by acoustic Doppler velocimeter. (**B**) Corresponding sensor measurement for three loading and unloading cycles.

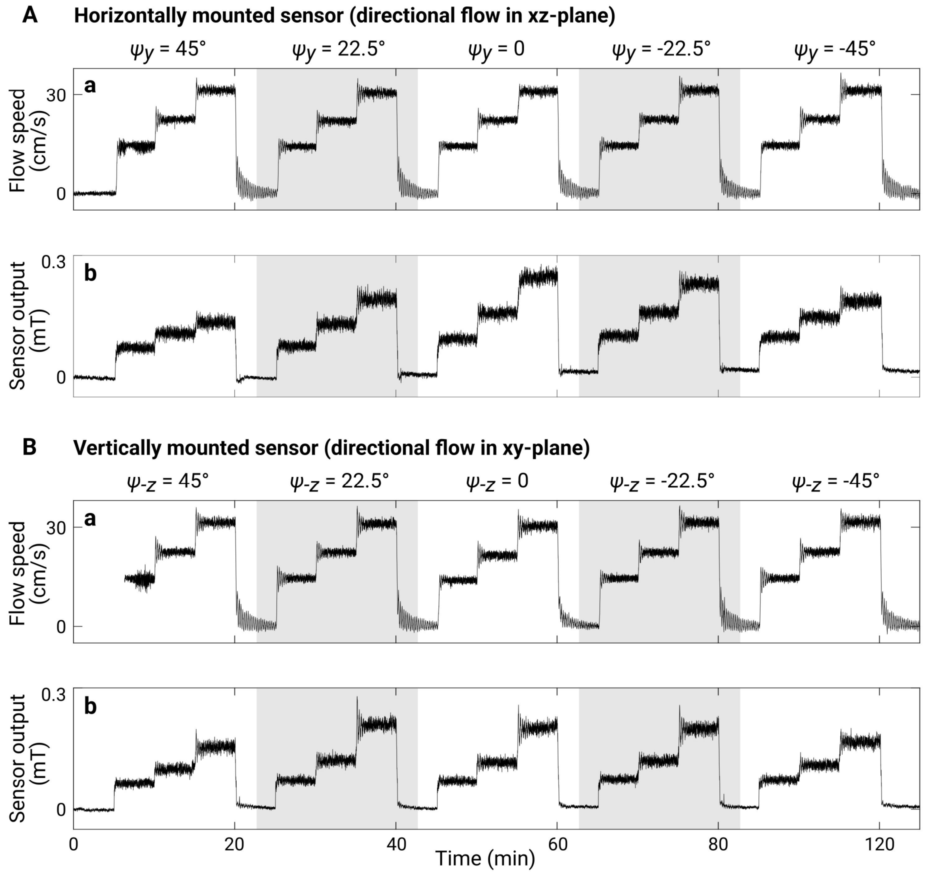

Fig. S3. Time series of orientation-dependent sensor response to directional flow.
(**A**) Directional flow velocity in *xz*-plane (a) and corresponding sensor output (b). (**B**) Directional flow velocity in *xy*-plane (a) and corresponding sensor output (b).

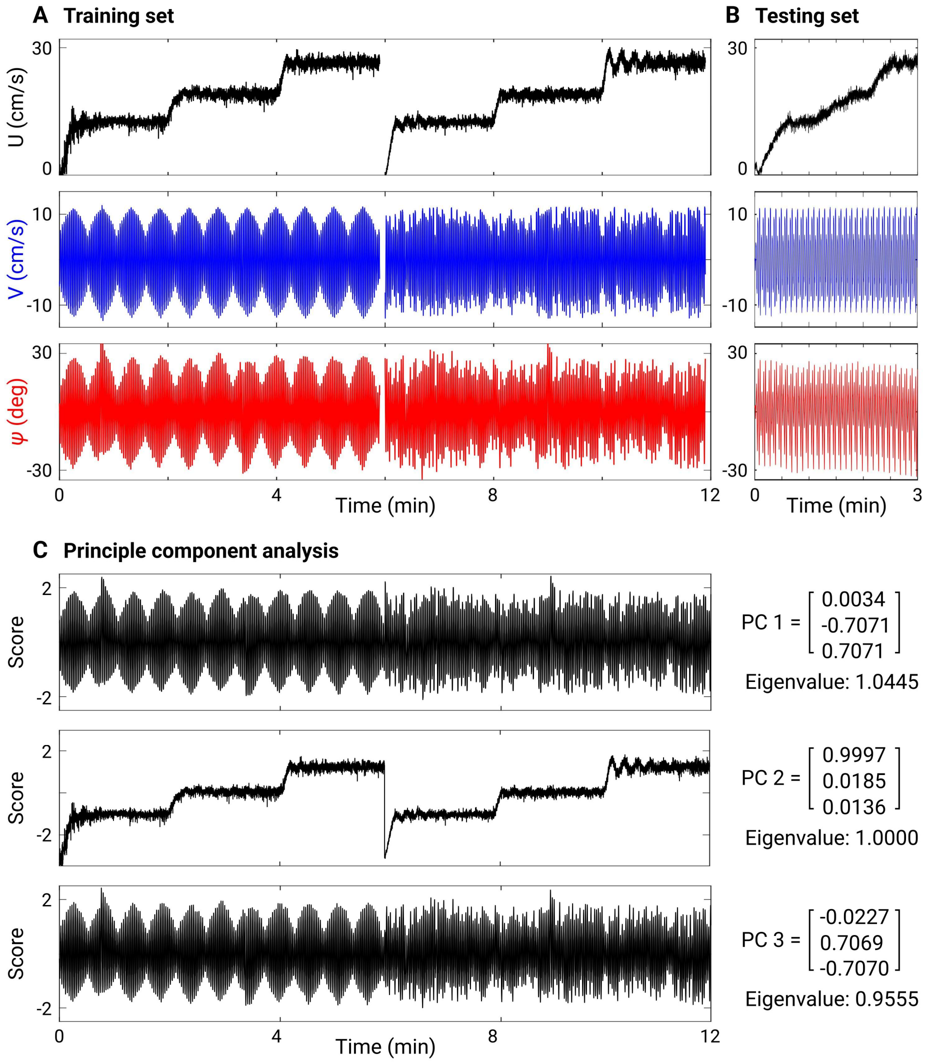

Fig. S4. Data used to train and test the model for estimating motion in uniform flow. (**A**) Training and (**B**) testing data. (**C**) Principal component analysis of the three states (U, V, and ψ) in the training set.

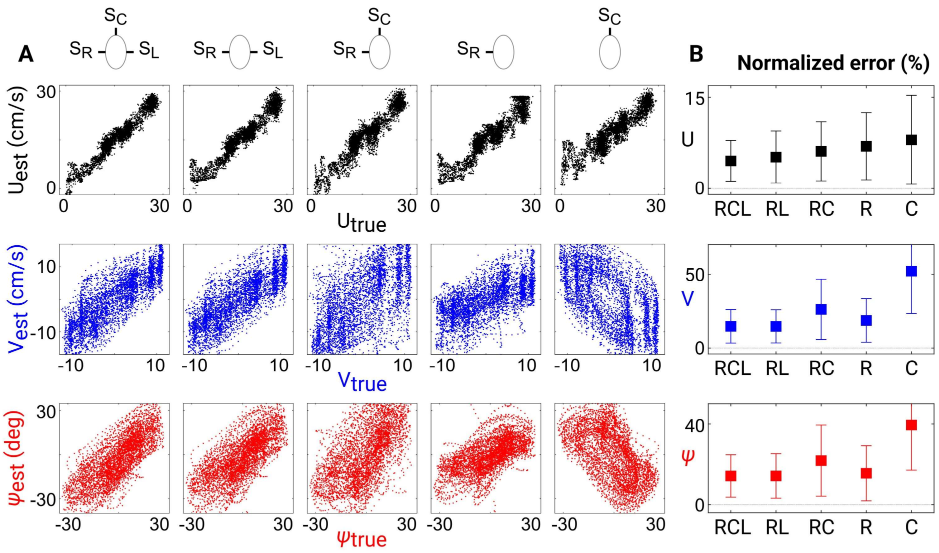

Fig. S5. Effect of number and placement of the sensors on estimation performance.
(**A**) Comparison between the true and estimated states (speed of the horizontal flow, U, lateral speed, V, and yaw angle, ψ, of the robot) using different numbers and placements of sensors. (**B**) Mean and standard deviation of absolute errors of the estimations normalized by the full-scale ranges of the states.

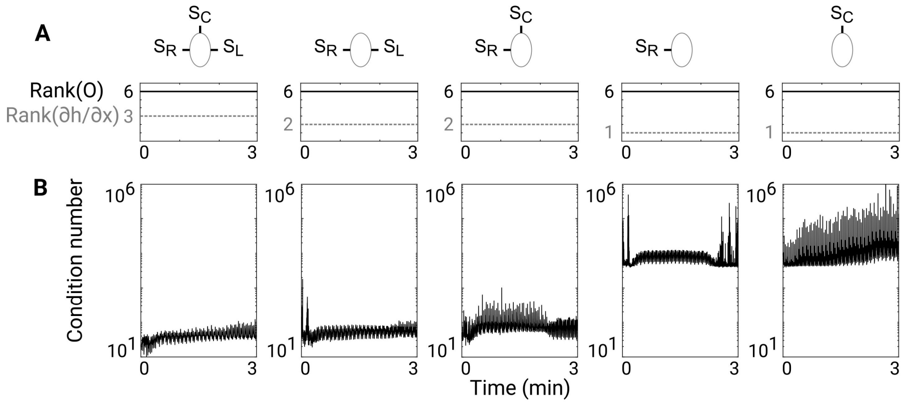

Fig. S6. Effect of number and placement of the sensors on observability.

(**A**) Ranks of the linearized measurement matrix and observability matrices. (**B**) Condition number of the observability matrices at each time step for different combinations of the sensors used in estimation with the extended Kalman filter.

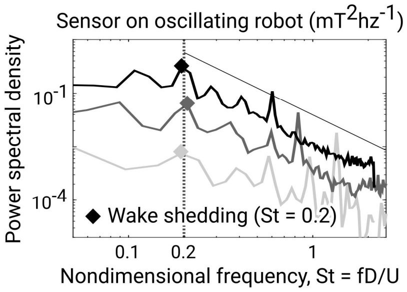

Fig. S7. Energy spectra measured by the sensor on the oscillating head, after removing the head oscillation component (*f* = 0.5 Hz) using a band-stop filter (stopband: 0.49–0.51 Hz).

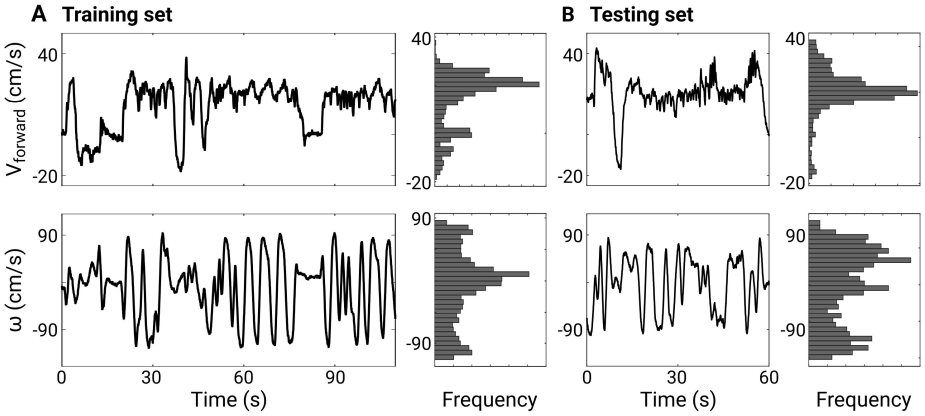

Fig. S8. Data used to train and test the model for estimating motion of free-swimming robotic fish in the pool.

Time series and histogram of the forward ($V_{forward}$) and angular (ω) velocities used as the training set (**A**) and testing set (**B**).

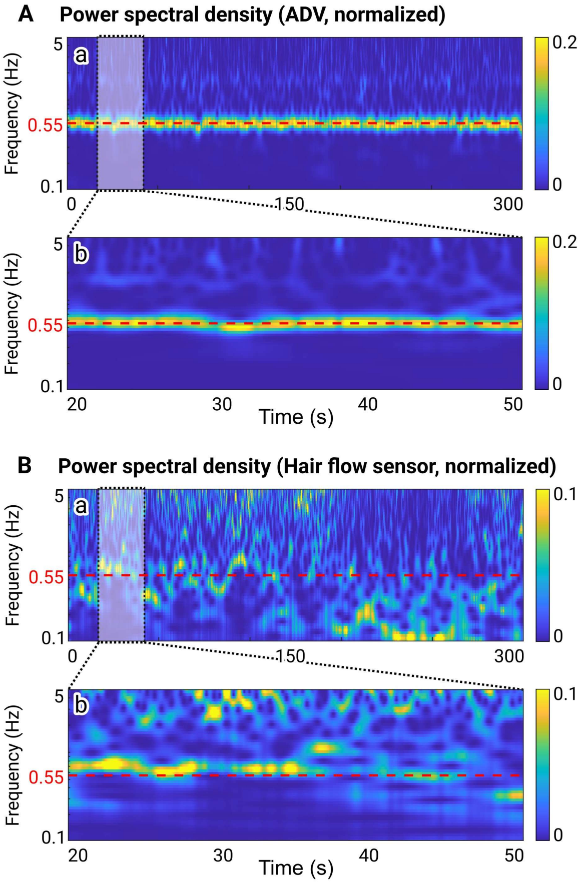

Fig. S9. Scalogram of measurements by acoustic Doppler velocimeter and hair flow sensor on free-swimming robotic fish behind a cylinder in water flume.

(**A**) Scalogram of ADV measurements of wake (with frequency of 0.55 Hz, indicated by the red horizontal dashed line) for 300 seconds (a) and for 30 seconds (b, See Movie S7 and accompanying caption). Colors represent per-time relative power spectral density (normalized by total power at each time) from continuous wavelet transform of the signals between 0.1 and 5 Hz. (**B**) Scalogram of the hair flow sensor measurements on the robotic fish swimming against the wake behind the cylinder for 300 seconds (a) and for 30 seconds (b).

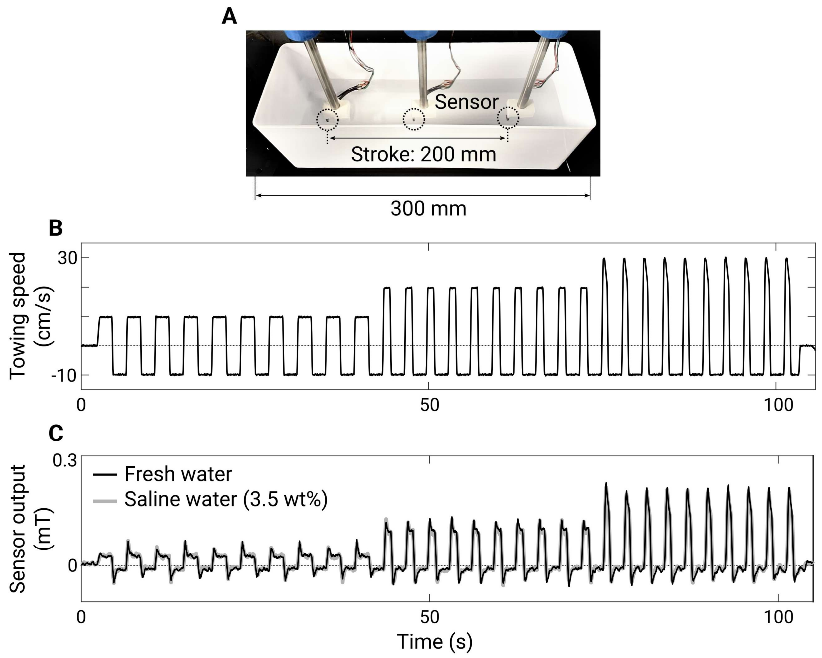

Fig. S10. Salinity effect on sensor response.

(**A**) Experimental setup for testing effect of salinity of water on sensor response. The sensor was towed by a robot arm at controlled speeds. (**B**) Time series of towing speed. (**C**) Time series of sensor outputs in fresh water and saline water.

Table S1.
Comparison of flow sensing technologies. Costs are in USD.

Device	Size	Cost	Sensitivity	Environmental dependency
Doppler velocity log (12—15)	Bulky (> 66 mm diameter)	High (>$10k)	High	Acoustic backscatter
Cameras	Compact	Low	Variable	Visibility
Pressure sensors (19—26)	Compact	Low	Variable	Depth, flow speed
Electromagnetic sensors (18)	Bulky (~700 mm length)	Moderate (< $5k)	High	Conductivity
Biomimetic sensors (27—52)	Compact to moderate (μm—cm)	N/A	Variable	Physical impact
Magnetic hair sensors (this work)	Compact (12 mm length)	Low (<$1)	Variable	Physical impact, magnetic disturbance

Movie S1. Free vibration of soft magnetic hair in air and water.

Comparison between free vibration of soft magnetic hair in air and water. In the water the hair was more damped than in the air.

Movie S2. Deflection of soft magnetic hair in directional flow in *xy*-plane.

Deflection of soft magnetic hair by flow at different speeds (15, 23, 30 cm/s) while changing its orientation in *xy*-plane (-45°, -22.5°, 0, 22.5°, 45°). Flow was horizontal and directed from right to left.

Movie S3. Oscillating motion of the soft underwater robot with three hair flow sensors.

Anterior part of a soft underwater robot with three flow sensors mounted on its head, oscillating at 0.5 Hz driven by a hydraulic bending actuator.

Movie S4. Visualization of true and estimated states of oscillating soft underwater robot in uniform flow.

Comparison between true and estimated horizontal flow speed (U, black arrows), lateral speed (V, blue arrows) and yaw angle (ψ) of the soft underwater robot for three oscillations (six seconds). Dotted lines indicate true states and solid lines indicate their estimations.

Movie S5. Hair flow sensors on oscillating mount in the wake behind a cylinder.

Oscillating head of a soft underwater robot with three hair flow sensors in the wake behind a cylinder. Alternating vortices were shed at Strouhal frequency (f = 0.55 Hz), which was deforming the magnetic hairs accordingly.

Movie S6. Visualization of true and estimated states of oscillating soft underwater robot in nonuniform flow.

Comparison between true and estimated horizontal flow speed ($\widetilde{U}_x$, black arrows), lateral speed (V, blue arrows) and yaw angle (ψ) of the soft underwater robot for three oscillations (six seconds). Dotted lines indicate true states and solid lines indicate their estimations.

Movie S7. Estimation of motion of free-swimming robotic fish.

A free-swimming robotic fish in the pool with three hair flow sensors installed on the bottom of its body (top row), outputs of the hair flow sensors to the forward and angular velocities during the swimming (middle row), and comparison between true and estimated velocities (Bottom row). Gray dotted curve and marker indicate true velocities and black solid curve and black marker indicate their estimations. Positive angular velocities indicate counterclockwise rotations of the robot.

Movie S8. Detection of flow structure near free-swimming robotic fish.

A robotic fish in the flume, swimming against the flow (upstream velocity: ~15 cm/s) in the wake behind a cylinder (bottom row) and wavelet spectra of measurement by hair flow sensors and acoustic Doppler velocimeter at each moment (top row). Prior to 40 seconds, the robotic fish was moving towards the cylinder, which caused the detected peak frequency to exceed the wake frequency (Doppler shift). Between 40 and 47 seconds, the fish remained stationary, and the sensor captured the exact frequency. After this period, when the fish began to sway significantly in the lateral direction, the detected frequency became less distinct and more difficult to clearly identify.
